# Observation and treatment in *DDX41*-mutated acute myeloid leukemia and myelodysplastic syndrome

**DOI:** 10.1038/s41408-023-00818-6

**Published:** 2023-04-10

**Authors:** Aref Al-Kali, Ahmad Nanaa, David Viswanatha, Rong He, Phuong Nguyen, Dragan Jevremovic, James M. Foran, Cecelia Arana Yi, Patricia T. Greipp, Naseema Gangat, Mrinal Patnaik, Ayalew Tefferi, Mark R. Litzow, Abhishek A. Mangaonkar, Mithun Vinod Shah, Talha Badar, Hassan B. Alkhateeb

**Affiliations:** 1 Division of Hematology, Mayo Clinic, Rochester, MN 55905 USA; 2 John H. Stroger, Jr. Hospital of Cook County, Chicago, IL 60612 USA; 3 Division of Hematopathology, Mayo Clinic, Rochester, MN 55905 USA; 4 Division of Hematology, Mayo Clinic, Jacksonville, FL 32224 USA; 5 Division of Hematology, Mayo Clinic, Scottsdale, AZ 85259 USA

**Keywords:** Cancer genetics, Cancer therapy

Dear Editor,

*DDX41*, a DEAD/H-box helicase gene located on chromosome 5q35.3, mutation (m) is rarely seen in myeloid neoplasms (1–2%) and is usually associated with myelodysplastic neoplasms (MDS) and acute myeloid leukemia (AML) [1–3]. It has been recently linked to more a favorable outcome despite its presentation with higher grade MDS and AML with higher response rates and long overall survival [4, 5]. Additionally, allogeneic hematopoietic cell transplantation (HCT), the only potentially curative option, has been linked to higher non-relapse mortality and therefore potentially to consider delayed HCT at disease progression or relapse [4, 6]. Some *DDX41* mutations can potentially be of germline origin and such work-up is warranted in these cases [7]. *mDDX41* MDS/AML cases tend to have indolent course, slow progression and higher hemoglobin and platelets indices [8]. In some of these cases watch and wait” (observation) approach can be attempted if the patient is stable with reasonable blood counts. We therefore report our single institution experience of “observation” vs active treatment and the outcome of both groups. We believe this is the first report to review this approach.

This is a single-institution retrospective study within Mayo Clinic Cancer Center (Rochester, Florida, Arizona). After Institutional Review Board approval, we screened, and chart reviewed m*DDX41* from up to 4524 consecutive Mayo Clinic patient samples submitted for 42-gene MN panel next generation sequencing (NGS) testing between 2018–2022. All NGS testing was done clinically, and results were available to treating physician prior to finalizing plan. m*DDX41* cases were included at diagnosis date and patients without pre-myeloid or myeloid neoplasias were excluded. Decision for observation was done when patient had acceptable hemoglobin (>10 gm/dL) and platelets count, while treatment was initiated when these parameters changed significantly. Germline analysis was available in some of the cases. All statistical analyses were performed using JMP® 16.2.0 Software (Supplemental methods).

We included forty patients with *DDX41* genetic alterations, of which 39 (97.5%) had at least one pathogenic *DDX41* mutation and one (2.5%) patient with a proven germline *DDX41-*VUS (Fig. 1A, B and Table S1). Twenty-seven (68%) patients received treatment with median time-to-treatment of 0.7 (0–3.2) months while 13 (32%) were observed; of which 9 (75%) received treatment later-on with median time-to-treatment of 16 (7.2–92) months (none of the thirteen patients died while under observation). The most common diagnosis in both groups was MDS (*N* = 14, 52% vs. *N* = 11, 85%; *p* = 0.045) and the second most common diagnosis was AML (*N* = 10, 37% vs. *N* = 1, 8%; *p* = 0.036) in the treatment group compared to the observation group; respectively. The majority of MDS patients in both groups were intermediate risk (64% vs. 55%; *p* = 0.6) per the revised international prognostic scoring system (IPSS-R), and MDS-EB-2 (excess blasts) (*N* = 10; 71% vs. *N* = 7; 64%) in the treatment and observation group; respectively. Only 3 AML patients in treatment group were adverse risk European Leukemia Net 2022 (ELN) and all other were intermediate-risk including one AML patient in observation group (Table 1).

**Fig. 1:**
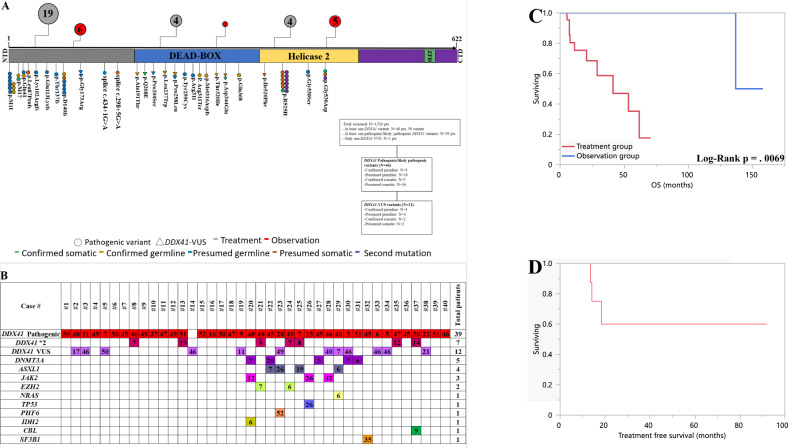
Observation and treatment in *DDX41* mutated myeloid neoplasms. **A** Distribution of *DDX41* variants detected, positioned on the *DDX41*protein and its functional domains with representation germline & somatic status and flowchart of the study. Number of patients in the treatment and observation group in relation to the predominate *DDX41* mutation location are represented in the red and grey circles. **B** Patterns of the co-mutations identified in the *DDX41* cohort and respective VAF value. **C** Kaplan–Meier survival curve in m*DDX41* patients grouped by treatment and observation group. **D** treatment-free-survival for observation cohort. NTD N-terminal domain, ZFD zinc finger domain, CTD C-terminal domain, Pts, patient; *2 Second pathogenic *DDX41* mutation.

**Table 1:** Characteristics and hematological features of mutated *DDX41* patients and comparison between treatment and observation group.

Variable	*DDX41* Treatment	*DDX41* Observation	*P* value
**No. of patients, (%)**	27 (68)	13 (32)	
**Age years, median (range)**	66 (53–81)	76 (50–88)	0.02*
**Sex (male),** ***n*** **(%)**	19 (70)	7 (54)	0.3
**Hemoglobin G/dL, median (range)**	10.5 (6.6–15.6)	11.9 (9.1– 14.9)	0.065
**Leukocytes 109/L, median (range)**	2.1 (1.3–8.5)	2.5 (1–5)	0.9
**Thrombocytes 109/L, median (range)**	90 (28–571)	124 (66–335)	0.036*
**ANC, median (range)**	0.95 (0.16–4.78)	0.95 (0.36–4.11)	0.7
**MCV median (range)**	104.2 (88.4–115)	107 (96–111)	0.6
**RDW, median (range)**	14.5 (12.5–23.4)	13.65 (12.6–17)	0.16
**BM blasts, median (range)**	15 (0–50)	11 (0–28)	0.15
**BM blasts (AML only), median (range)**	30 (20–50)	28	0.8
**Number of co-mutations, median (range)**	0 (0–3)	0 (0–1)	0.5
**Isolated** ***DDX41*** **mutations,** ***n*** **(%)**	17 (63)	9 (70)	0.7
***DDX41*** **VAF, median (range)**	46.5 (6–51)	45 (5–52)	0.7
**Several pathogenic** ***DDX41*** **mutations in single case,** ***n*** **(%)**	5 (19)	2 (17)	0.85
**Dominant mutation type,** ***n*** **(%)**			
Missense	4 (15)	6 (46)	0.038*
Nonsense	3 (12)	2 (15.5)	0.7
Frameshift	9 (35)	2 (15.5)	0.1
Splice site mutation	2 (8)	0	0.3
Start-loss variant	8 (30)	3(23)	0.6
**Diagnosis,** ***n*** **(%)**			
MDS	14 (52)	11 (85)	0.045*
-MDS-EB-2	10 (71)	7 (64)	–
-MDS-EB-1	1 (7)	0	–
AML	10 (37)	1 (8)	0.036*
MPN	2 (7)	0	–
CCUS	1 (4)	1 (8)	–
**IPSS-R,** ***n*** **(%)**			
**Low**	2 (14.3)	3 (27)	0.4
**Intermediate**	9 (64.3)	6 (55)	0.6
**High**	3 (21.4)	2 (18)	0.8
**Abnormal cytogenetics,** ***n*** **(%)**	1 (4)	2 (15)	0.2

*ANC* absolute neutrophil count, *MCV* mean corpuscular volume, *RDW* red cell distribution width, *BM* bone marrow, *VAF* variant allele frequency, *MDS* myelodysplasia neoplasm, *AML* acute myeloid leukemia, *MPN* myeloproliferative neoplasms, *CCUS* clonal cytopenia of undetermined significance, *VUS* variants of unknown significance.

*Statistically significant.

The median age at diagnosis for treatment and observation group was 66 and 76 years, respectively (*p* = 0.2) and both groups had more males (*N* = 19; 70% vs. *N* = 7; 54%; *p* = 0.7). Observation group had higher blood counts; however, this was only statistically significant for platelet count (124 vs. 90; *p* = 0.036). Bone marrow blast count was higher in the treatment group compared to the observation group (for both overall cohort and AML patients) (*p* = .15, *p* = 0.8; respectively). Most patients in both groups had normal cytogenetics (*N* = 26; 96% vs. *N* = 11; 85%; *p* = 0.2) and none of our patients had a complex karyotype. The most common mutation type in the observation group was missense mutation (46% vs. 15%; *p* = 0.04), on the other hand a frameshift mutation was the most common mutation type in the treatment group (35% vs. 15.5%; *p* = 0.1) (Table 1 and Table S1). The most common dominant variant in the observation group was p.R525H (5; 39%) and p.M1I was the most common in the treatment group (7; 27%) and the second common variant in both cohorts was p. Asp140Glyfs (*N* = 4; 16% vs. *N* = 2; 15%). Although five patients in the treatment group had a second *DDX41*(p.R525H) mutations.

The median number of co-mutations in each group was zero and the median *DDX41* VAF was (47% vs. 45%; *p* = 0.7). The majority of patients had isolated-*DDX41* mutation (*N* = 9; 70% vs. *N* = 17; 63%; *p* = 0.7) and the most common co-mutation in the observation group was *DNMT3A* (15%), and *ASXL1* (15%) was the most common in treatment group. Of thirty-nine patients with pathogenic *DDX41*-varient, 7 (18%) patients had a second *DDX41* mutation and 11 (28%) had a *DDX41*-VUS (Fig. 1B, Table 1, Table S1, and Fig. S1). Overall, 15 (38%) patients had germline testing (13 patients were proven to harbor at least one germline *DDX41*-varient and two patients were proven to be somatic variants). Six (46%) patients in the observation arm were tested, of which 5 confirmed to be germline and one was somatic (Fig. 1A and Table S1).

Overall, twelve (44%) and one (8%) patient died in the treatment and observation group respectively after a median follow-up of 43 months. None died within 60 days in either group. We performed survival analysis on MDS/AML sub-group for homogeneity purpose (we excluded one MDS patient from the analysis, who was lost to follow up within 1.5 months) and found significantly better OS in observation group compared to treatment group with 5-year OS of 100% in observation group compared to median OS of 41 months in treatment group (*p* = 0.0069) (Fig. 1C). Repeating the same analysis in MDS patients showed similar results (*p* = 0.008) (Fig. S2).

In the observation group 2 (18%) patients of the MDS group (both harboring p.R525H variant) progressed into AML (only one was treated at time of progression) with median time-to-progression of 20 months compared to 4 (29%) in the treatment group with median time-to-progression of 11.7 months (*p* = 0.05). In the treatment MDS/AML group 16 patients received hematopoietic stem cell transplantation (HSCT); of whom 7 (44%) died.

Median treatment-free-survival (TFS, time observed until initiation of treatment or death) for observation group was not reached and 1-year estimated (YE) TFS was 100% (5YE-TFS was 60%) (Fig. 1D). In the MDS/AML observation arm, eight patients subsequently received treatment, 3 achieved complete remission (CR), 1 partial remission (PR), 2 hematological improvement (HI), and 2 did not respond (NR). Of the 2 MDS patients who did not respond to first treatment, they received additional treatment with hypomethylating agent (HMA) after which one patient achieved PR, and 1 NR. Of the 2 MDS patients who progressed to AML under observation, one patient started on HMA + Venetoclax (VEN) therapy after progression, while the other one received induction with Daunorubicin/Cytarabine 24 months after progression. All three patients who did not receive any treatment are still alive and the CCUS patient received treatment with erythropoietin stimulating agent (ESA). In the treatment MDS/AML arm 12 patients received HMA based regimen, 6 received chemotherapy, 1 ESA, and 5 HMA + VEN; of which 16 achieved CR, 1 PR, and 7 NR with a median time to response from treatment initiation of 3.1 months. Two MPN patients died, both initially received hydroxyurea and one received ruxolitinib then HSCT (Tables S3–4). The CCUS patient received ESA, then progressed to MDS and received HMA. All patients who received HMA + VEN or chemotherapy achieved complete remission.

We hereby report the possible option of “observing” m*DDX41* MDS/AML patients with close to normal hematopoiesis, behaving as an indolent myeloid neoplasm despite the presence of high-grade blasts. We also show the safety of this option, as none died during observation or after starting treatment when indicated. The overall survival of the observation group was favorable with no patients dying within the first five years. We note for the first time that “observation” group had a significantly increased presence of missense mutation (especially R525H) which could be an escape rescue mechanism for hematopoiesis. *DDX41* gene is essential for protein synthesis and monoallelic *DDX41* mutations may not affect hematopoiesis like biallelic mutations do; therefore, functional studies are needed in this group of patients [9]. Finally, all of our patients responded to HMA + VEN therapy, which is a novel finding. Our paper is limited by the smaller size, retrospective nature, from a single institution, and the lack of germline analysis in all cases. We believe however, that our findings are important for future work and collaboration on this rare genetic abnormality.

## Supplementary information

Supplementary material

## Data Availability

Gene panel sequencing data are available by request to the corresponding author at Alkali.aref@mayo.edu
