# Supplementary material for: Observation and treatment in DDX41-mutated acute myeloid leukemia and myelodysplastic syndrome
Source: Blood Cancer J. 2023 Apr 10;13(1):49. doi: 10.1038/s41408-023-00818-6 (PMC10083167; doi:10.1038/s41408-023-00818-6)
Supplement: Supplementary file 1 — Supplementary material [file 41408_2023_818_MOESM1_ESM.pdf]

Supplementary

Observation and treatment in *DDX41*-mutated acute myelogenous leukemia and myelodysplastic syndrome

Aref Al-Kali[1*], Ahmad Nanaa[1,5*], David Viswanatha[2], Rong He[2], Phuong Nguyen[2], Dragan Jevremovic[2], James M. Foran[3], Cecelia Arana Yi[4], Patricia T Greipp[2], Naseema Gangat[1], Mrinal Patnaik[1], Ayalew Tefferi[1], Mark R Litzow[1], Abhishek A. Mangaonkar[1], Mithun Vinod Shah[1], Talha Badar[3], Hassan B Alkhateeb[1]

[1] Division of Hematology, Mayo Clinic, Rochester, MN 55905, USA

[2] Division of Hematopathology, Mayo Clinic, Rochester, MN 55905, USA

[3] Division of Hematology, Mayo Clinic, Jacksonville, FL 32224, USA

[4] Division of Hematology, Mayo Clinic, Scottsdale, AZ 85259, USA

[5] John H. Stroger, Jr. Hospital of Cook County, Chicago, IL 60612, USA

Supplemental methods

This is a retrospective, single institution study within the Mayo Clinic (Rochester, Florida, Arizona). We retrospectively screened up to 4,524 consecutive patients who had next-generation sequencing (NGS) testing on peripheral blood or bone marrow specimens (OncoHeme, Mayo Clinic) between 2018-2022. Chart review of mutated *DDX41* cases was done after approval by the Institutional Review Board (IRB). The procedures followed were in accordance with the Declaration of Helsinki of 1975, as revised in the year 2000. In this study we included patients with pathogenic-*DDX41* mutation and one patient with proven germline *DDX41* variants of unknown significance (VUS), all patients were included at diagnosis date, and we excluded patients without pre-myeloid or myeloid neoplasias. we excluded one MDS patient from the analysis, who was lost to follow up within 1.5 months. Patients with more than one pathogenic/likely pathogenic mutations detected in the same gene, the mutation with highest variant allele frequency (VAF) value was included in the analysis only. Bone marrow biopsy and aspirate were reviewed by a hematopathologist as a part of a routine clinical practice. The diagnosis was rendered according to World Health Organization (WHO) 2016 classification of myeloid neoplasms. [1, 2]. Germline testing was performed using either a skin fibroblast culture or sorted T-cell testing. Fifteen *DDX41* patients got germline testing, of which 13 identified as having germline alteration and 2 as having somatic *DDX41* mutations. All patients except 6 (15%) had *DDX41* variant with VAF value higher than 40%.

Mutation analysis and next generation sequencing

OncoHeme, Mayo Clinic NGS was performed on peripheral blood or bone marrow specimens and the panel included 42 genes: *ASXL1, BCOR, CALR, CBL, CEBPA, NPM1, NRAS, PHF6, PTPN11, RUNX1, SETBP1, SF3B1, TERT, TET2, TP53, U2AF1, WT1, CSF3R, DNMT3A, ETV6, EZH2, FLT3, GATA1, GATA2, IDH1, IDH2, JAK2, KIT, KRAS, MPL, MYD88, ZRSR2, ANKRD26, DDX41, ELANE, ETNK1, KDM6A, RAD21, SH2B3, SRP72, SMC3, and STAG2*.[3] DNA was target-enriched with a custom hybridization-capture reagent (SureSelectXT, Agilent, Santa Clara, CA) and sequenced on the HiSeq platforms (Illumina, San Diego, CA) at the Mayo Clinic Clinical Genome Sequencing Laboratory. Sequencing data was processed through a custom bioinformatics pipeline (Mayo NGS Workbench), using CLC Bio Genomics Server v6.0 (Qiagen, Redwood City, CA) for alignment and variant calling. The aligned BAM files were further processed through an in-house developed break point algorithm for larger insertion/deletion detection (>25 bp). BAM files of all variant calls were manually reviewed in the genome browser Alamut® Visual (Interactive Biosoftware, Rouen, France) for confirmation. The limit of detection of the NGS assay is 5% with a minimum 250X depth of coverage. More than 95% of tested regions had >1000X depth of coverage in the clinical assay. Genetic variants were curated and annotated in the Mayo Clinic Molecular Hematopathology Laboratory following guidelines from the American College of Medical Genetics and Genomics (ACMG) five-tier system.[4]

Statistical analyses

All analyses were performed using JMP® 16.2.0 Software. Comparisons of categorical parameters were performed using the Chi-square test ($\chi 2$). For continuous variables, Wilcoxon test was used. Overall survival (OS) estimates were calculated using Kaplan-Meier curves which were calculated from diagnosis date and the date of death due to any cause. Patients who were alive at the last follow-up date were censored at that time. Competing risk analysis to calculate treatment-free-survival (TFS) was measured from diagnosis date until either death or starting treatment, whichever occurred first. To ameliorate the influence of hematopoietic stem cell transplantation (HSCT) on survival, TFS and OS were censored at the time of HSCT in patients who received HSCT. P values $< .05$ were considered statistically significant. Response to treatment was assessed based on the International Working Group criteria[5, 6.]

Table S1. Molecular features of forty *DDX41* mutated patients.

Case ID #	Isolated (ISO), Co-mutated (CO)	Nucleotide variant	Amino acid change	Mutation type	VAF (%)	Germline status of patients	*DDX41*- variants of unknown significance (VAF%)	Myeloid neoplasm presentation	Cytogenetics	Co-mutation
D1*	ISO	c.3G>A	p.M1I	start-loss variant	50	CG		MDS	46, XY [20]	
D2*	ISO	c.3G>A	p.M1I	start-loss variant	48	CG	c.1558A>T; p.Ile520Phe (17%)	AML	46, XX [20]	
D3*	ISO	c.1574G>A	p.Arg525His	Missense	11	CG	c.773C>T; p.Pro258Leu (46%)	AML	46, XY [20].	
D4	ISO	c.3G>A	p.M1I	start-loss variant	49	PG		AML	46, XY [20]	
D5	ISO	c.1574G>A	p.Arg525His	Missense	7	PG	c.517G>A; p.Gly173Arg (50%)	MDS	Normal	
D6	ISO	c.3G>A	p.M1I	start-loss variant	50	PG		MDS	46, XX [20]	
D7	ISO	c.415_418dup	p.Asp140Glyfs	Frameshift	45	PG		MDS	46, XY [20]	
D8	ISO	c.305_306del, c.1574G>A	p.Lys102Argfs*32, p. Arg525His	Frameshift, Missense	46, 7	PG		AML	46, XY [20]	
D9*	ISO	c.1102C>T	p.Gln368*	Nonsense	49	CG		MDS	46, XY [20]	
D10	ISO	c.415_418dup	p.Asp140Glyfs	Frameshift	37	PS		MDS	47, XY, +Y [2]/46, XY [18]	
D11	ISO	c.3G>A	p.M1I	start-loss variant	47	PG		AML in remission	46, XY [20]	
D12	ISO	c.3G>A	p.M1I	start-loss variant	49	PG		MDS	Normal	
D13	ISO	c.776A>G, c.1574G>A	p.Tyr259Cys, p.Arg525His	Missense, Missense	51, 19	PG		MDS	46, XY [20]	
D14*	ISO					CG	c.710T>G; p.Leu237Trp (46%)	MDS	46, XY [20]	
D15	ISO	c.3G>A	p.M1I	start-loss variant	52	PG		AML	46, XY [20]	
D16	ISO	c.1574G>A	p.Arg525His	Missense	16	PS		MDS	46, XY [20].	
D17	ISO	c.3G>A	p.M1I	start-loss variant	50	PG		MDS	46, XY [20]	
D18*	ISO	c.946_947del	p.Met316Aspfs*31	Frameshift	47	CG		MDS	45, X, -Y [5]46, XY [15]	

**D19**	**ISO**	**c.1574G>A**	**p.Arg525His**	**Missense**	**9**	**CS**	**c.622C>G (p.Q208E) (11%)**	**MDS**	**46, XY [21]**	
**D20**	**CO**	**c.337del**	**p.Glu113Lysfs*14**	**Frameshift**	**48**	**PG**		**MPN**	**46,XY,del(20)(q11.2q 13.1)[15]/4 6,XY[5]**	***DNMT3A*, *IDH2*, *JAK2***
**D21**	**CO**	**c.931C>T, c.1574G>A**	**p.Arg311*, p.Arg525His**	**Nonsense, Missense**	**48, 8**	**PG**		**AML**	**46, XX [20]**	***EZH2***
**D22**	**CO**	**c.415_418dup**	**p. Asp140Glyfs**	**Frameshift**	**45**	**PG**		**AML**	**46,XY[20]**	***ASXL1*, *DNMT3A***
**D23***	**CO**	**c.1589G>A**	**p. Gly530Asp**	**Missense**	**28**	**CG**	**c.959C>T; p.Thr320Ile (49%)**	**AML**	**46, XY [20]**	***ASXL1*, *PHF6***
**D24***	**CO**	**c.3G>A, c.1574G>A**	**p.M1I, p. Arg525His**	**start-loss variant, Missense**	**49, 7**	**CG**		**MDS**	**46, XY [20].**	***EZH2***
**D25**	**CO**	**c.260del, c.1574G>A**	**p. Leu87Profs*24, p. Arg525His**	**Missense, Missense**	**7, 8**	**PS**		**MDS**	**46, XX [20]**	***ASXL1***
**D26**	**CO**	**c.298+5G>A**	**p.?**	**splice site mutation**	**15**	**PS**		**MPN**	**Normal**	***JAK2*, *TP53***
**D27***	**CO**	**c.415_418dup**	**p. Asp140Glyfs**	**Frameshift**	**45**	**CG**		**MDS**	**46, XX, del (5) (q13q22), del (20) (q11.2q13. 3) [14]/46, XX [6]**	***DNMT3A***
**D28**	**CO**	**c.1589G>A**	**p. Gly530Asp**	**Missense**	**46**	**PG**	**c.610C>T; p.Pro204Ser (40%)**	**MDS**	**46,XY[20]**	***JAK2***
**D29**	**CO**	**c.434+1G>A**	**p.?**	**splice site mutation**	**46**	**PG**	**c.1032C>G; p.Asp344Glu (7%)**	**MDS**	**46, XX [20].**	***ASXL1*, *NRAS***
**D30**	**CO**	**c.1574G>A**	**p. Arg525His**	**Missense**	**5**	**PG**	**c.517G>A; p.Gly173Arg (46%)**	**CCUS**	**47, XY, +8[14]/46, XY [6]**	***DNMT3A***
**D31**	**CO**	**c.121C>T**	**p. Gln41***	**Nonsense**	**51**	**PG**		**AML**		***DNMT3A***
**D32**	**CO**	**c.415_418dup**	**p. Asp140Glyfs**	**Frameshift**	**45**	**PG**		**MDS**	**46, XX [20]**	***SF3B1***
**D33**	**ISO**	**c.1588G>A**	**p. Gly530Ser**	**Missense**	**6**	**PG**	**c.773C>T; p.Pro258Leu (46%)**	**MDS**	**46,XY[20]**	
**D34***	**ISO**	**c.1574G>A**	**p. Arg525His**	**Missense**	**5**	**CG**	**c.571G>A; p.Ala191Thr (46%)**	**MDS**	**46, XX**	
**D35**	**ISO**	**c.409dup, c.1574G>A**	**p. Thr137fs, p. Arg525His**	**Frameshift, Missense**	**47, 12**	**PG**		**AML**	**46, XX**	

**D36***	**ISO**	**c.931C>T**	**P. Arg311Ter**	**Nonsense**	**45**	**CG**		**MDS**	**46,XX**	
**D37***	**CO**	**c.1A>C, 1589G>A**	**p.M1? p. Gly530Asp**	**start-loss variant, Missense**	**50, 14**	**CG**		**MDS**	**46,XX**	***CBL***
**D38**	**ISO**	**c.1A>C**	**p.M1?**	**start-loss variant**	**22**	**CS**	**c.1032C>G; p.Asp344Glu (21%)**	**CCUS**	**46,XX[20]**	
**D39***	**ISO**	**c.121C>T**	**p. Gln41***	**Nonsense**	**51**	**CG**		**MDS**	**46,XX[20]**	
**D40**	**ISO**	**c.415_418dup**	**p. Asp140Glyfs**	**Frameshift**	**46**	**PG**		**MDS**	**46,XY[20]**	

Abbreviations: D, *DDX41* mutated patients; *, Proven germline; C, confirmed; P, presumed; G, germline; S, somatic; MDS, myelodysplasia neoplasm; AML, acute myeloid leukemia; MPN, Myeloproliferative neoplasms; CCUS, clonal cytopenia of undetermined significance; Iso, Isolated; Co, Co-mutated; VAF, variant allele frequencies.

Table S2. Co-mutation frequency and variant allele frequencies distribution in *DDX41* co-mutated patients.

Gene	N	Median, %	Range
DNMT3A	5	7	5-20
ASXL1	4	13	6-26
JAK2	3	26	12-42
EZH2	2	7	6-7
NRAS	1	6	
TP53	1	26	
PHF6	1	52	
IDH2	1	6	
CBL	1	9	
SF3B1	1	35	

Table S3. Treatment one regimens and outcomes in *DDX41* mutated MDS/AML patients (Treatment arm)

**Treatment**	**Received treatment, N (%)**	**Responder, N (RR%)**
HMA	12 (50)	6 (50)
Chemotherapy	6 (25)	6 (100)
HMA plus Venetoclax	5 (21)	5 (100)
ESAs	1 (4)	0 (0)

Table S4. Treatment one regimens and outcomes in *DDX41* mutated MDS/AML patients (Observation arm)

**Treatment**	**Received treatment, N (%)**	**Responder, N (RR%)**
HMA	1 (12.5)	0 (0)
Chemotherapy	1 (12.5)	1 (100)
HMA plus Venetoclax	3 (37.5)	3 (100)
lenalidomide	1 (12.5)	1 (100)
ESAs	2 (25)	1 (50)

Abbreviations: RR, Response Rate; HMA, hypomethylating agents; ESAs, Erythropoiesis-stimulating agents.

Figure S1. Patterns of the co-mutations identified in *DDX41* MDS only patients and respective VAF value in treatment (T) and observation group (O).

Case #	#5O	#13O	#16O	#17O	#19O	#27O	#32O	#34O	#36O	#37O	#39O	#1T	#6T	#7T	#9T	#10T	#12T	#14T	#18T	#24T	#25T	#28T	#29T	#33T	#40T
***DDX41* Pathogenic**	7	51	16	50	9	45	45	5	45	50	51	50	50	45	49	37	49		47	49	7	46	46	6	46
***DDX41* *2**		19								14										7	8				
***DDX41* VUS**	50				11			46										46				40	7	46	
DNMT3A						5																			
ASXL1																					19		6		
JAK2																						42			
EZH2																				6					
NRAS																							6		
CBL										9															
SF3B1							35																		

Figure S2. Kaplan–Meier survival curve in m*DDX41* MDS patients grouped by treatment and observation group.

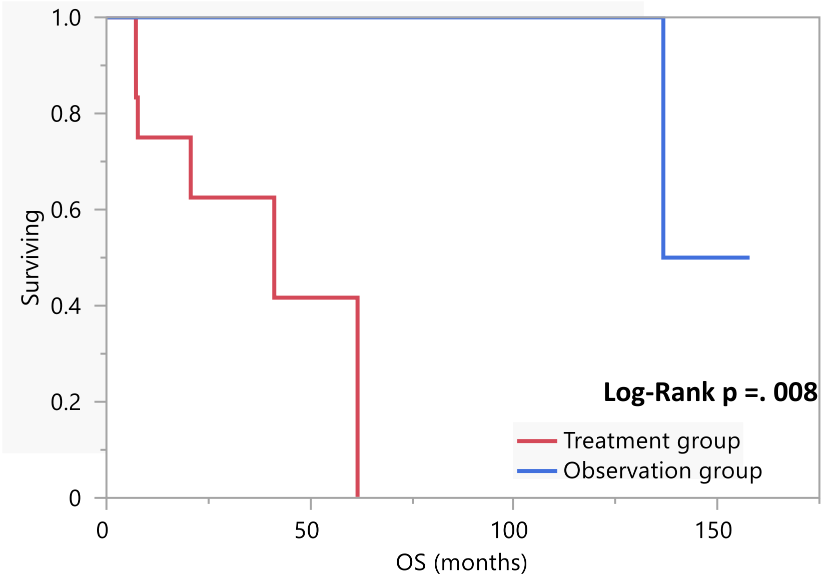

References

1. Arber DA, Orazi A, Hasserjian R, Thiele J, Borowitz MJ, Le Beau MM, et al. The 2016 revision to the World Health Organization classification of myeloid neoplasms and acute leukemia. Blood. 2016;127(20):2391-405.
2. Vardiman JW, Harris NL, Brunning RD. The World Health Organization (WHO) classification of the myeloid neoplasms. Blood. 2002;100(7):2292-302.
3. He R, Devine DJ, Tu ZJ, Mai M, Chen D, Nguyen PL, et al. Hybridization capture-based next generation sequencing reliably detects FLT3 mutations and classifies FLT3-internal tandem duplication allelic ratio in acute myeloid leukemia: a comparative study to standard fragment analysis. Modern Pathology. 2020;33(3):334-43.
4. Richards S, Aziz N, Bale S, Bick D, Das S, Gastier-Foster J, et al. Standards and guidelines for the interpretation of sequence variants: a joint consensus recommendation of the American College of Medical Genetics and Genomics and the Association for Molecular Pathology. Genetics in Medicine. 2015;17(5):405-23.

5. Cheson BD, Bennett JM, Kopecky KJ, Büchner T, Willman CL, Estey EH, et al. Revised recommendations of the International Working Group for Diagnosis, Standardization of Response Criteria, Treatment Outcomes, and Reporting Standards for Therapeutic Trials in Acute Myeloid Leukemia. J Clin Oncol. 2003;21(24):4642-9.

6. Cheson BD, Greenberg PL, Bennett JM, Lowenberg B, Wijermans PW, Nimer SD, et al. Clinical application and proposal for modification of the International Working Group (IWG) response criteria in myelodysplasia. Blood. 2006;108(2):419-25.
